# Epidural abscess formation after chemotherapy for breast cancer: a case report and literature review

**DOI:** 10.3389/fsurg.2025.1388278

**Published:** 2025-01-23

**Authors:** Youzhi An, Lili Li, Qingning Liu, Zhen Zhang, Xuelin Lin

**Affiliations:** ^1^ Second Department of Spinal Surgery, The Second People’s Hospital of Liaocheng, The Second Hospital of Liaocheng Affiliated to Shandong First Medical University, Linqing, Shandong, China; ^2^ Medical Oncology, The Second People’s Hospital of Liaocheng, The Second Hospital of Liaocheng Affiliated to Shandong First Medical University, Linqing, Shandong, China

**Keywords:** epidural abscess, case report, breast cancer, chemotherapy, complication

## Abstract

**Introduction:**

Spinal epidural abscess is a rare infectious lesion of the central nervous system. Here, we report a rare case of a thoracic suppurative epidural abscess in a female patient who developed incomplete paralysis of both lower limbs after chemotherapy for breast cancer. She underwent surgery and recovered well after surgery.

**Case report:**

A 49-year-old female patient developed an epidural abscess after chemotherapy for breast cancer; she suffered sudden pain and paralysis in both lower limbs. Thoracic T9–T11 laminectomy, abscess removal, bone grafting, fusion, and internal fixation were performed. After the operation, the muscle strength in both lower limbs gradually recovered.

**Discussion:**

This is the first reported case of an epidural abscess after chemotherapy for breast cancer. The disease progresses rapidly. During the literature review process, we found that timely removal of the epidural abscess, combined with the administration of appropriate antibiotics at the same time, is crucial for improved healing and successful treatment.

## Introduction

Spinal epidural abscess (SEA) is a rare infectious lesion of the central nervous system with high rates of disability and mortality. It may take only a few days from the onset of symptoms to the occurrence of irreversible neurological deficits. Therefore, early diagnosis and timely treatment are crucial for the management of spinal epidural abscess. We reviewed the literature and found reports of spinal epidural abscess; however, cases of spinal epidural abscess after chemotherapy for breast cancer have not been documented. We retrospectively analyzed a case of thoracic suppurative epidural abscess with incomplete paralysis of both lower limbs after chemotherapy for breast cancer. After active surgery and comprehensive treatment, the outcome was satisfactory. The details of the case report are below.

## Case report

**Figure 1:**
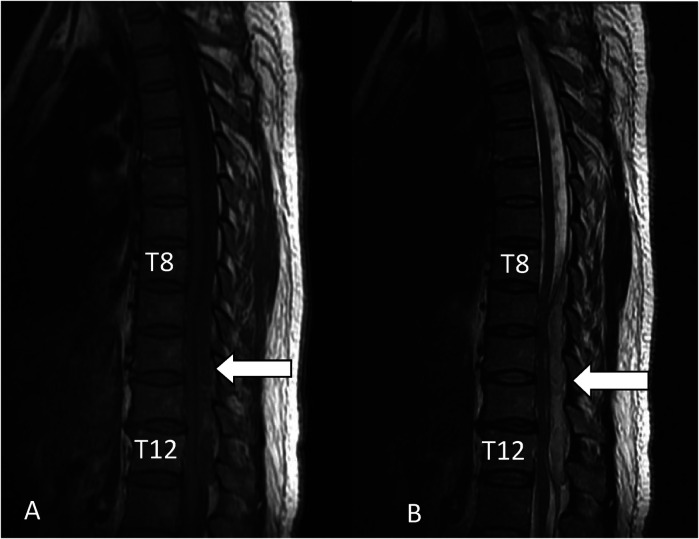
Intraspinal epidural abscess from the posterior T8 to T12 vertebral body. (**A**) Sagittal T1-weighted MRI showing SEA (white arrowhead). (**B**) Sagittal T2-weighted MRI showing SEA (white arrowhead).

The patient is a 49-year-old married female farmer with a history of good physical and mental health. She has no family history of genetic diseases or chronic medical conditions. Five months ago, she underwent a radical mastectomy at our hospital, followed by eight cycles of chemotherapy. She experienced mild myelosuppression and leukopenia after chemotherapy. She was admitted to the Radiotherapy Department of our hospital with the main complaint of being “more than 5 months after breast cancer surgery and 1 week after the last chemotherapy for radiotherapy.”. After admission, a routine blood test revealed a white blood cell count of 1.61 × 10^9^/L and a neutrophil ratio of 17.4%. Consequently, measures were taken to raise her white blood cell count and enhance her immunity in preparation for radiotherapy. On day 13, the patient developed severe burning pain in the left lower abdomen and the front of the thigh, accompanied by abdominal distension and poor appetite; her body temperature remained normal, with no tenderness in the chest and back; sensation and muscle strength in both lower limbs were normal; she was treated with analgesics. However, on the morning of day 16, the patient suddenly experienced weakness in both lower limbs, became unable to stand, and reported chest and back pain. An urgent thoracic spine magnetic resonance imaging (MRI) showed longitudinal and extramedullary epidural strips, with mixed signal shadows of slightly longer T2 and short and long T1 in the horizontal spinal canal of the T8–T12 vertebrae; the signals were poorly defined and uneven, measuring approximately 120 mm × 14 mm × 10 mm. The findings included anterior compression of the dural sac and spinal cord, spinal canal stenosis, and lamellar strips with slightly longer T2 signal shadows and poorly defined boundaries. The following diagnosis was made: T8–T12 spinal canal space with spinal edema and degeneration, highly suggestive of an epidural abscess (Figure 1). Urgent routine blood and C-reactive protein tests revealed a white blood cell count of 13.48 × 10^9^/L, a neutrophil ratio of 83.9%, a lymphocyte ratio of 9.3%, a red blood cell count of 2.61 × 10^12^/L, a hemoglobin level of 82 g/L, and a C-reactive protein (quantitative) level of 105.25 mg/L. Physical examination revealed normal body temperature, chest and back tenderness, decreased sensation below the umbilicus, normal muscle strength in both upper limbs, grade 2 muscle strength in both lower limbs, slightly decreased muscle tension in both lower limbs, numbness in the perineal and perianal areas, absence of bilateral knee and Achilles tendon reflexes, and negative Babinski and Hoffman signs. She was then transferred to our department for further treatment.

After being transferred to our department, the patient received an infusion of two units of red blood cells. On day 17, thoracic T9–T11 laminectomy, abscess removal, bone grafting, fusion, and internal fixation were performed. Intraoperative findings included a yellow-white viscous substance in the spinal canal from T9 to T11; part of the substance was sent for pathological examination, bacterial culture, and drug sensitivity testing. After the suction was completed, the area was repeatedly washed with a large amount of diluted iodine and normal saline until the rinse solution was clean and free of turbidity. The removed posterior wall of the spinal canal was soaked in diluted iodophor; we then removed it from the iodophor and trimmed it into bone granules; we implanted the constructed bone granules into the bilateral facet joints of T9–T12.

**Figure 2:**
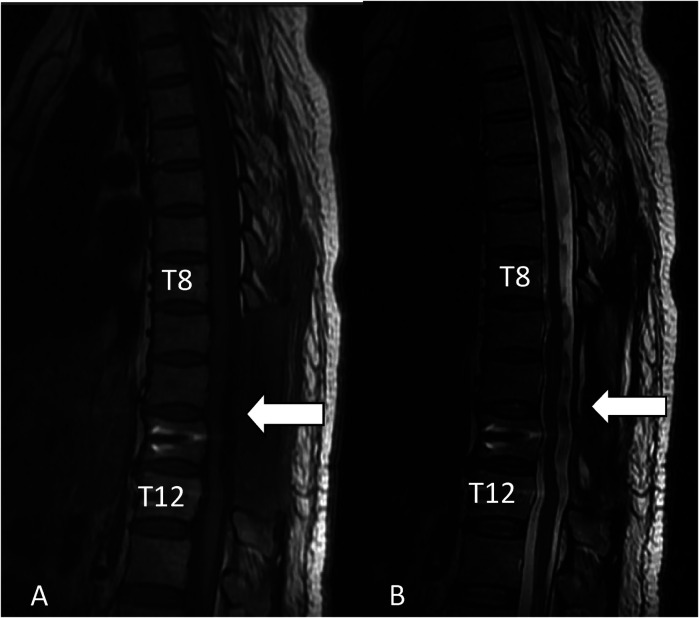
Sagittal T1-weighted MRI showing that the epidural abscess has been cleared and decompressed adequately (white arrowhead), and the sagittal T2-weighted MRI showing that the epidural abscess has been cleared and decompressed adequately (white arrowhead).

Postoperative pathological results showed a mass in the spinal canal, infiltration of a large number of inflammatory cells, and abscess formation. Bacterial culture findings identified *Staphylococcus aureus*, with a colony count of +++. Drug sensitivity results revealed that all strains were sensitive to oxacillin, gentamicin, cefazolin, levofloxacin, clindamycin, and vancomycin. Cefazolin sodium pentahydrate was given intravenously for 2 weeks after the operation. The patient's abdominal distension and lower abdominal pain were significantly relieved on the first postoperative day; on the second postoperative day, routine blood and C-reactive protein examination showed the following: white blood cell count 8.73 × 10^9^/L, neutrophils 77.5%, lymphocytes 15.5%, red blood cell count 3.80 × 10^12^/L, hemoglobin 114 g/L, and C-reactive protein 25.08 mg/L; the muscle strength in both lower limbs improved to grade 3. There was no tenderness in the chest and back, and the numbness in the perineal and perianal areas was significantly relieved compared to that before the operation. With the help of family members, she could sit, stand, and walk while wearing a tight chest brace. After discharge, she was prescribed oral levofloxacin for 10 weeks to ensure complete elimination of bacteria and to prevent recurrence. One month after the operation, she was returned to the hospital for a follow-up thoracic MRI (Figure 2), which showed no compression of the dura and the absence of an abscess.

Physical examination showed that the surgical incision had healed well, with no tenderness in the chest and back, normal sensation and muscle strength in both lower limbs, normal stool, and urine output, normal muscle tension in both lower limbs, normal knee and Achilles tendon reflexes, and negative Babinski and Hoffman signs.

## Discussion

SEA is a rare but serious infectious disease that carries a high rate of morbidity and mortality (1). It is a suppurative infection of the spine that occurs in the epidural space between the dura mater and the vertebral periosteum (2–4). Risk factors for SEA include diabetes mellitus, bacteremia, chronic steroid use, and contiguous infection. SEA after chemoradiation therapy for esophageal cancer has been reported (5). To the best of our knowledge, our case is the first report of spinal epidural abscess formation after breast cancer chemotherapy in the English literature.

Myelosuppression generally appears 7–10 days after chemotherapy. The patient was admitted to the hospital 1 week after chemotherapy, and routine blood tests revealed that the white blood cell and neutrophil counts were significantly lower than normal levels, which was considered severe myelosuppression, likely compounded by the previous chemotherapy history of the patient. The patient's neutrophil count was 0.28  ×  10^9^/L, indicating a state of granulocytopenia. She had no other causes of immunodeficiency other than chemotherapy after breast cancer surgery. At this time, the patient's immune system was extremely compromised, making her highly susceptible to infection. When infections do occur, they are usually severe. The most common clinical sites of infection include the respiratory tract, urinary system, blood system, and digestive system. In this case, an MRI examination was performed because of weakness in both lower limbs, which revealed an occupying lesion in the spinal canal, which was confirmed to be a local abscess by a pathology; the causative pathogen was *S. aureus*. The patient had no history of local trauma or diabetes, and it was thought that the infection resulted from the direct entry of bacteria into the bloodstream due to reduced body resistance after granulocytosis. The infection site was identified as an epidural abscess. The incidence of myelosuppression combined with infection at this site after chemotherapy is very low and rare in clinical practice, which is worthy of attention and reference. If not detected timely and treated actively, it may lead to irreversible complications.

The classic triad of back pain, fever, and neurologic deficit occurs in less than 8% of cases (1). The patient's initial symptom was severe radiating nerve root pain, without fever or back pain. Over the course of 4 days, the root pain in the lower limbs progressed to weakness and paralysis. The rapid progression of symptoms made an appropriate and prompt diagnosis of an epidural abscess quite challenging (6).

Spinal MRI is the gold standard for diagnosing SEA. If clinicians are unaware that SEA can develop in breast cancer patients undergoing chemotherapy, the spinal MRI may be delayed. A delay in diagnosis is associated with an increased risk of residual neurologic deficits (7, 8). Early surgery is indicated for the majority of patients, especially those with developing or worsening neurologic deficits (9, 10). Surgical treatment within 24 h is associated with improved outcomes (11). SEA causes compression of the spinal cord, resulting in acute spinal cord injury. Surgery is recommended for acute spinal cord injury within 24 h (12). Maintaining adequate perfusion pressure and mean arterial pressure after acute spinal cord injury is beneficial for nerve recovery (13). Intraoperative ultrasound offers significant advantages in evaluating the adequacy of decompression, particularly in cases of thoracic ossification of the posterior longitudinal ligament (14). The epidural abscess was almost entirely on the dorsal side of the dura, and it was successfully cleared under direct visualization, with good dural pulsation and satisfactory spinal cord decompression after abscess clearance.

Therefore, we did not use intraoperative ultrasound again to assess the adequacy of decompression.

In conclusion, an epidural abscess is a rare but life-threatening complication that can develop after chemotherapy for breast cancer. Once SEA is suspected, an immediate MRI examination is essential; if diagnosed, surgical decompression is often needed. Early diagnosis, rapid surgical decompression, and sensitive antibiotic therapy are the keys to successful treatment.

## Data Availability

The original contributions presented in the study are included in the article/Supplementary Material; further inquiries can be directed to the corresponding authors.
